# Biosecurity risks for African swine fever occurrence in smallholder systems in Gauteng, South Africa

**DOI:** 10.4102/ojvr.v93i1.2235

**Published:** 2026-05-15

**Authors:** Keneiloe P. Montsu, Catharina I. Boshoff, Eric Etter, Juanita van Emmenes

**Affiliations:** 1 Transboundary Animal Disease, Onderstepoort Veterinary Research, Agricultural Research Council, Onderstepoort, South Africa; 2 Department of Biomedical Sciences, Faculty of Science, Tshwane University of Technology, Pretoria, South Africa; 3 Animal, Health, Territories, Risks, and Ecosystems (ASTRE), University of Montpellier, Montpellier, France; 4 Center for International Cooperation in Agronomic Research for Development (CIRAD), Guadeloupe, France; 5 Department of Production Animal Studies, Faculty of Veterinary Sciences, University of Pretoria, Pretoria, South Africa

**Keywords:** African swine fever, biosecurity, risk, Gauteng, farmers, South Africa

## Abstract

African swine fever (ASF) outbreaks in Gauteng province (2012, 2019–2022) occurred outside South Africa’s (SA’s) traditional control zones and highlighted biosecurity deficiencies among smallholder producers. This study aimed to assess biosecurity practices and knowledge gaps among smallholder pig farmers in Gauteng by comparing ASF-free areas with high-risk areas (HRA) that had or had not experienced ASF outbreaks. Between 2020 and 2021, structured interviews were conducted with 137 pig farmers across four municipalities: Ekurhuleni, Lesedi, Tshwane and West Rand. The questionnaire captured data on farm and household characteristics, herd size and ownership, animal health management, biosecurity practices, pig movement and trade, ASF awareness, and potential routes of virus introduction. Farmers in HRA should implement additional biosecurity measures. Key risk factors included the absence of quarantine facilities (*p* < 0.05), improper carcass disposal and limited knowledge of ASF transmission.

**Contribution:**

The findings provide baseline data crucial for developing evidence-based ASF control strategies targeting SA’s peri-urban smallholder pig production systems.

## Introduction

African swine fever (ASF) is a highly infectious viral disease affecting pigs. It was first reported as an acute haemorrhagic fever in East Africa in the early 1900s (Dixon et al. 2020; Montgomery 1921). The virus infects domestic pigs and wild suids such as bush pigs or warthogs and *Ornithodoros* species ticks. Warthogs and soft ticks maintain ASF virus (ASFV) through the sylvatic cycle, which does not cause any pathogenic effects in these hosts. Understanding these dynamics is essential for managing and preventing outbreaks in naïve pig populations (Van Rensburg et al. 2020b). Direct transmission of ASFV occurs between infected and susceptible pigs, whereas indirect transmission occurs when pigs encounter contaminated food, bedding and utensils (Brown & Bevins 2018).

African swine fever is a controlled disease in South Africa (SA) in terms of *the Animal Diseases Act, 1984 (Act 35 of 1984)*, and has been reported to the World Organisation for Animal Health (WOAH). The Department of Agriculture (DOA), which was previously known as the Department of Agriculture, Forestry and Fisheries (DAFF), has produced a detailed veterinary procedural notice (VPN) standard for ASF control, which clearly outlines control of ASF. The control area in SA was instituted in 1935 in Limpopo, northern parts of North West and KwaZulu-Natal and north-eastern parts of Mpumalanga, as indicated in *the Animal Diseases Act of South Africa (Act 35 of 1984)*. A control zone is defined within a part of the country to distinguish between infected and disease-free zones, and this is done to progressively control diseases and allow trade in livestock and their commodities (Fujita 2004). Pig production in SA is practised in all nine provinces, with Limpopo, North West, Gauteng and KwaZulu-Natal having the highest numbers of pig farmers (Munzhelele, Oguttu & Fasina 2016). According to Stats SA (2016), in Gauteng province, agricultural households that farm with a combination of livestock (cattle, sheep, goats, pigs and chicken) make up only 29.0% of all farmers, and 1.1% of these households are engaged in a combination of pig and poultry farming.

*The Animal Diseases Act, 1984 (Act 35 of 1984)* was initially proven effective for SA in controlling ASF within the control zone. However, since 2016 ASF outbreaks in domestic pigs have not been linked to the recent transfer of infected animals or materials from within the controlled area (Van Rensburg et al. 2020b). Outbreaks of ASF outside the control zone cause significant losses to farmers because of its high mortality rate, trade restrictions and severe socio-economic impact (Van Rensburg et al. 2020b). According to Penrith (2020), smallholder and backyard farms provide conditions that facilitate ASFV circulation. The control of ASF includes biosecurity measures and movement control, but challenges remain when it comes to smallholder (subsistence) farmers, as they do not always have the financial means and understanding to effectively implement the biosecurity measures that will aid in the prevention and control of ASF (Dione et al. 2020).

The effects of ASF infection in the pig industry are overwhelming because they restrict trade and production, whereas for smallholder farmers in developing countries, livelihoods are affected as they use pigs as income sources (Sánchez-Cordón et al. 2018). Minimum standards for accredited piggeries should include access control (fencing, personnel and visitors, vehicle access, animal access), internal biosecurity, pest control, feed and feed quality, a farm plan, care and management, transport (loading, vehicle and vehicle hygiene) and records for new pig introductions as well as pig movements (DAFF 2018). The objective of this study focuses on providing specific, local data on biosecurity gaps and knowledge deficits regarding ASF in Gauteng. This is the first systematic biosecurity assessment in Gauteng province, SA’s most economically important and densely populated region. Gauteng’s peri-urban production landscape – characterised by high population density, market integration, informal settlements and complex urban-rural interfaces – differs from the rural Limpopo settings of prior SA studies (Fasina et al. 2015; Mokoele et al. 2014) and East African contexts. These contextual differences necessitate location-specific assessment rather than generalisability.

## Research methods and design

### Study area

**FIGURE 1:**
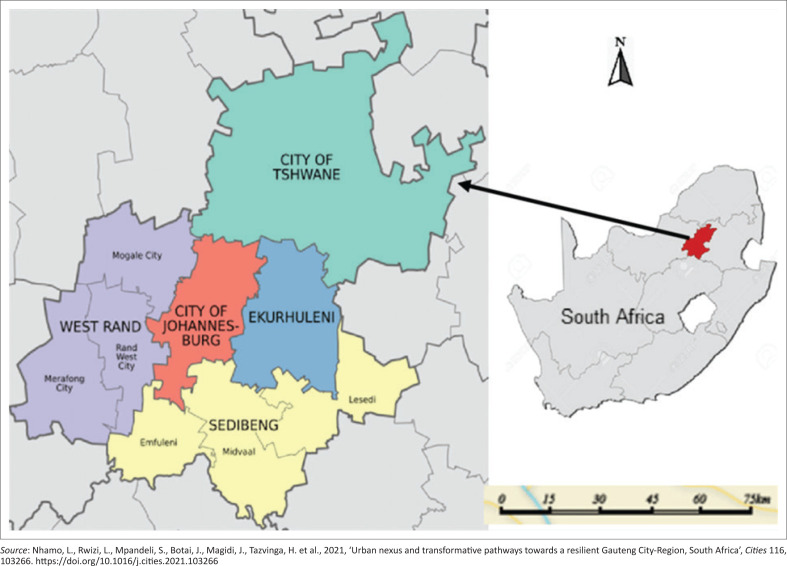
Map of Gauteng province indicating the four sampling municipal districts Ekurhuleni (Blue), Lesedi (Yellow), Tshwane (Green), and West rand (Purple).

This study was conducted in Gauteng province, SA. Extension officers from the Gauteng Department of Agriculture and Rural Development (GDARD) collaborated by facilitating introductions to the smallholder pig farmers in their respective regions. Farmers from four municipal districts: Ekurhuleni, Lesedi, Tshwane and West Rand (Figure 1) were included in the database. These districts were selected based on their history of ASF occurrence or their ASF-free status at the time of the study. The four municipalities were classified as either high-risk or ASF-free zones, with Tshwane municipality designated as ASF-free because of the absence of any recorded ASF outbreaks. The remaining three municipalities (Ekurhuleni, Lesedi, and West Rand) were grouped and classified as high-risk areas (HRA) because they had previously reported ASF outbreaks. Within the HRA, farms were further categorised into two groups: those that had experienced ASF outbreaks and those that had not.

### Sample size

The snowball sampling approach targeted individuals in Gauteng who were potentially exposed to ASF, primarily those with low biosecurity practices. A conservative approach was selected to maximise the sample size. This sampling technique facilitated the inclusion of participants sharing similar social conditions and experiences. For the sample size calculations, the expected population proportion (*p*) was assumed to be 10% (*p* = 0.10). A 95% confidence level with a desired absolute precision (*e*) of 5% was applied. The formula used was adapted from Cochran (1963) and Thrusfield (2005): *n* = (*Z*^2^ × *P* × (1 – *P*))/*e*^2^; where *n* = required sample size, *Z* = value from the standard normal distribution corresponding to the desired confidence level (*Z* = 1.96 for 95% confidence interval), *P* = expected population proportion, and *e* = desired precision. Applying this formula, the required sample size for this study was approximately 139 respondents.

### Data collection

Structured questionnaires were used to collect information from farmers or pig owners (Asambe, Sackey & Tedek 2019). The questionnaires were conducted in English. During farmers’ days or meetings, smallholder farmers who were willing to participate in the study were recruited after being informed about the project and obtaining their informed consent. Because of the coronavirus disease 2019 (COVID-19) pandemic, the study was supplemented by a method of calling farmers, as most of the participants could not be reached as a result of the COVID-19 restriction protocols. The questionnaire was divided into seven sections: (1) farmer demographics; (2) livestock ownership; (3) pig and animal health management; (4) biosecurity measures; (5) pig trade and movement; (6) awareness of ASF; and (7) possible virus introduction modes.

### Data analysis

Questionnaire data were recorded using Excel spreadsheets. All responses were entered into a spreadsheet, and the raw data were then organised and cleaned for subsequent analysis. The raw data were screened for any errors that might have occurred during entry, and errors were corrected by rechecking the original questionnaires. Excel pivot tables were used to generate tables and graphs to aid in analysis (Brown, Pan & Wiersma 2015). Proportions between the distinct categories (ASF-free-, HRA with no ASF reported [HRA {-} ASF] and HRA with previous ASF experience [HRA {+} ASF]) were compared using a non-parametric test known as Pearson’s Chi-square test (χ^2^), which was calculated using Statistical Analysis System (SAS, SAS Institute). The chi-squared test indicated a significant difference in each of the variables between the groups. Statistical significance (association) was set at *p* < 0.05. In addition, multiple comparison testing was performed. The Bonferroni correction was used, where the original α level is divided by the number of tests performed to get a new, stricter threshold for each test. False Discovery Rate (FDR) controls were performed to determine the proportion of ‘discoveries’ (meaningful results) that are false positives, rather than the probability of making even one false positive. Associations between biosecurity practices and ASF risk status were assessed using Pearson’s chi-square test or Fisher’s exact test where expected cell frequencies were less than five. Statistical significance was set at α = 0.05. Given the large number of comparisons performed (31 tests), we applied both Bonferroni correction (adjusted α = 0.05/31 = 0.0016) and FDR correction using the Benjamini-Hochberg procedure to control for Type I error inflation. Results are reported with both unadjusted and adjusted *p*-values to allow assessment of statistical significance under different correction stringencies.

### Ethical considerations

Ethical clearance to conduct this study was obtained from Tshwane University of Technology and Faculty Committee for Research Ethics-Science (No. FCRE 2021/01/003 [FCPS 02] [SCI]).

## Results

A total of 137 farmers were interviewed across the Gauteng study areas (27 from ASF-free areas + 110 from HRA = 137 total). Notably, participation was significantly lower in ASF-free areas compared to HRA: 27/137 (19.7%) versus 110/137 (80.3%), *p* < 0.05.

### Participants’ demographics

Of the participants, 69 (50%) were older than 50 years, 49 (36%) were between 40 and 50, 11 (8%) were younger than 30 years and 8 (6%) did not report their age. Most respondents were male (*n* = 88, 64%), while females were (*n* = 49) 36%. The responses were classified into three categories: ASF-free areas (*n* = 27), HRA with no ASF reports (*n* = 93), and HRA with ASF reports (*n* = 17). This corresponds to 19.7% of respondents from ASF-free areas, 67.9% from HRA without ASF, and 12.4% from HRA where ASF was reported. Most responses originated from HRA without ASF occurrence, suggesting greater engagement or representation from regions under surveillance but not yet affected by ASF outbreaks.

### Pig population distribution

Across ASF status categories, ASF-free and HRA (–) ASF areas contained proportionally more piglets (56.3% and 48.8%, respectively), while HRA (+) ASF areas had higher proportions of sows (53.6%), fattening pigs (23%) and boars (4.6%) (Table 1), possibly reflecting herd recovery and restocking efforts following ASF-related losses. These variations were statistically significant (χ^2^ = 521.67–2246.28, *p* < 0.001), confirming that herd structure and population dynamics differ between municipalities and ASF risk zones.

**TABLE 1:** Total number of pigs: Sows, boars, fattening, and piglets.

ASF areas	ASF free (%)	HRA (-) ASF (%)	HRA (+) ASF (%)	χ^2^	*p*-value
Sows	24.0	26.5	53.6	521.67	< 0.001
Boars	2.5	4.0	4.6	146.77	< 0.001
Fattening	17.2	20.8	23.0	692.89	< 0.001
Piglets	56.3	48.8	18.8	2246.28	< 0.001

Note: *χ*^2^ Pearson’s Chi-square test; *p* < 0.05 indicates a statistically significant difference; *p* > 0.05 indicates no significant difference.

ASF, African swine fever; HRA, high-risk area.

### Pig housing and management practices

Pig management practices differed slightly across the three groups (Table 2). Most respondents in all areas kept pigs permanently in pens, with proportions of 92.6%, 95.7%, and 100% for ASF-free, HRA without ASF experience, and HRA with ASF experience, respectively (*p* = 0.504). Seasonal or free-roaming systems were rare, indicating a general preference for confined management.

**TABLE 2:** Pig management and health practices in African swine fever free and high-risk areas.

Variable	ASF free		HRA no ASF		HRA ASF		χ^2^	*p*-value
	*n*	%	*n*	%	*n*	%		
Permanently kept in pens	25	92.6	89	95.7	17	100.0	1.37	0.504
Free roaming in daytime, in pens at night	1	3.7	3	3.2	0	0.0	0.60	0.741
Free roaming	0	0.0	1	1.1	0	0.0	0.48	0.788
Seasonally free roaming	1	3.7	0	0.0	0	0.0	4.10	0.128
Health problems	5	18.5	46	49.5	10	58.8	9.72	0.008
Routine cleaning	27	100	90	96.8	17	100.0	1.45	0.484
Permission to move pigs	11	40.7	35	37.6	7	41.2	0.14	0.934
Disinfectant footbath	17	63.0	65	69.9	14	82.3	1.87	0.392
Washing hands	18	66.7	25	26.9	7	41.2	14.47	0.001
Change boots/clothes	13	48.1	19	20.4	4	23.5	8.37	0.015
Spray	6	22.2	6	6.5	2	11.8	5.72	0.057
Drive-through disinfectant	2	7.4	6	6.5	1	5.9	0.05	0.977
Wait 24 h before visiting another pig site	6	22.2	10	10.8	1	5.9	3.29	0.193

Note: Sum of percentages may exceed 100% where multiple responses were possible. The variable is indicated as a YES response; χ^2^ Pearson’s Chi-square test; *p* < 0.05 indicates a statistically significant difference; *p* > 0.05 indicates no significant difference.

ASF, African swine fever; HRA, high-risk area.

Significant variation was observed in reported pig health problems over the previous 6 months (χ^2^ = 9.72, *p* = 0.008), with higher reports from farmers in HRA with ASF experience (58.8%) compared to ASF-free areas (18.5%). Routine cleaning was widely practised across groups (> 96%), while obtaining official permission for pig movement varied slightly (37% – 41%) but was not statistically significant (*p* = 0.934). Biosecurity measures at pig facilities showed notable differences. Practices such as washing hands (*p* = 0.001) and changing boots and clothes (*p* = 0.015) were significantly more common among respondents with ASF experience. Conversely, the use of disinfectant footbaths and tyre baths did not differ significantly across groups. When managing sick pigs (Table 3), contacting a veterinarian was the most common action, reported by all respondents in ASF-free areas (100%), compared with 82.6% in HRA without ASF experience and 94.1% in HRA with ASF experience. The differences were statistically significant (*p* = 0.04), although the trend indicates a more consistent veterinary contact rate among respondents from ASF-free areas.

**TABLE 3:** Handling of sick pigs in African swine fever free and high-risk areas.

Variable	ASF free		HRA no ASF		HRA ASF		χ^2^	*p*-value
	*n*	%	*n*	%	*n*	%		
Contact Veterinarian	27	100.0	77	82.6	16	94.1	6.46	0.040
Sell the pigs	0	0.0	0	0.0	0	0.0	N/A	N/A
Slaughter at home	1	4.3	7	7.6	1	5.9	0.51	0.774
Send for slaughtering	0	0.0	1	1.1	1	5.9	2.81	0.246
Purchase more pigs	0	0.0	2	2.2	1	5.9	1.69	0.430
No sick pigs yet	0	0.0	4	4.3	0	0.0	1.95	0.377
Self-treat	0	0.0	5	5.4	0	0.0	2.46	0.293

Note: Sum of percentages may exceed 100% where multiple responses were possible. The variable is indicated as a YES response; χ^2^ Pearson’s Chi-square test; *p* < 0.05 indicates a statistically significant difference; *p* > 0.05 indicates no significant difference.

ASF, African swine fever; HRA, high-risk area; N/A, not applicable.

Home slaughtering was relatively low across all regions (4% – 8%), with no significant differences (*p* = 0.774). The low percentage of responses to pigs being slaughtered is reported as herd recovery and restocking efforts following ASF-related losses. Few respondents reported purchasing new pigs when existing pigs were sick (*p* = 0.43). Notably, a small proportion of farmers in HRA without ASF experience reported self-treatment of sick pigs (5.4%), which was not observed in other groups.

Methods of carcass disposal showed moderate variation between regions (Table 4). Burial was the most common disposal method in HRA (65.6% for no-ASF and 100% for ASF-experienced farms) compared to 50% in ASF-free areas. Burning of carcasses was reported by 38.5% of ASF-free farmers, 28% of HRA without ASF, and 5.9% of ASF-experienced farms (*p* = 0.26). Improper practices such as on-premises disposal, feeding carcasses to other pigs or selling/consuming infected meat were rarely reported. None of the groups admitted to selling or consuming carcasses, indicating a generally good understanding of ASF biosecurity risks.

**TABLE 4:** Handling of dead pigs in African swine fever free and high-risk areas.

Variable	ASF free		HRA no ASF		HRA ASF		χ^2^	*p*-value
	*n*	%	*n*	%	*n*	%		
Burn	10	38.5	26	28.0	1	5.9	5.27	0.072
Bury	14	50.0	61	65.6	17	100.0	11.28	0.004
Dispose of on premises	1	3.8	1	1.1	0	0.0	1.29	0.524
Dispose of on another site	1	3.8	1	1.1	0	0.0	1.29	0.524
Give as feed to other pigs	0	0.0	2	2.2	0	0.0	0.96	0.619
Sell the meat	0	0.0	0	0.0	0	0.0	N/A	N/A
Consume the meat	0	0.0	0	0.0	0	0.0	N/A	N/A
Give away the meat	1	3.8	1	1.1	0	0.0	1.29	0.524
No dead pig yet	0	0.0	1	1.1	0	0.0	0.48	0.788

Note: Sum of percentages may exceed 100% where multiple responses were possible. The variable is indicated as a YES response; χ^2^ Pearson’s Chi-square test; *p* < 0.05 indicates a statistically significant difference; *p* > 0.05 indicates no significant difference.

ASF, African swine fever; HRA, high-risk area; N/A, not applicable.

Supplementary analysis (Online Appendix 1) was conducted and can be summarised as follows: The total tests conducted were 31 (excluding N/A), the tests with *p* < 0.05 had 9 (29.0%) of the variables in Table 2-OA1, Table 3-OA1, and Table 4-OA1 significant. The multiple comparison adjustment (Online Appendix 1, Table 1-OA1) reveals a critical limitation of the original analysis. Of the 9 findings reported as statistically significant at *p* < 0.05 originally, only 5–6 remain significant after appropriate correction for multiple testing. The differences in herd composition across ASF status categories remain highly significant (all *p* < 0.001). This is unsurprising given the extremely large chi-square values (χ^2^ = 146–2246), suggesting genuine and substantial differences in herd structure. Handwashing practices (Bonferroni = 0.031, FDR = 0.008) represent the most robust behavioural difference between groups. African swine fever-free farmers (66.7%) wash hands significantly more frequently than HRA farmers without ASF experience (26.9%).

The burial of dead pigs (FDR = 0.031) was significant using both Chi-square and FDR correction but not the more conservative Bonferroni correction (*p* = 0.124). This borderline finding suggests farmers with ASF experience are more likely to bury carcasses (100% vs. 65.6% vs. 50%), although the evidence is weaker. However, contacting a veterinarian (Chi-square *p* = 0.040 → Bonferroni correction *p* = 1.000) is not statistically significant after correction. The apparent difference (100% vs. 82.6% vs. 94.1%) may be because of chance. Another behavioural measure that loses significance is the changing of boots or clothes (Chi-square *p* = 0.015 vs. Bonferroni correction *p* = 0.465), suggesting the observed pattern (48.1% vs. 20.4% vs. 23.5%) is not reliably different across groups. The higher rate of health problems in HRA with ASF (58.8%) compared to ASF-free areas (18.5%) becomes non-significant (Chi-square *p* = 0.008 vs. Bonferroni correction *p* = 0.248), although it approaches significance with FDR correction (FDR = 0.050).

## Discussion

The significant difference in the sample composition between ASF-free areas and HRA aligns with the established behavioural theory regarding leverage-salience and survey participation (Groves, Singer & Corning 2000). Several factors may contribute to this disparity: stakeholders in disease-free areas may perceive ASF-related surveys as less applicable to their immediate circumstances, reducing motivation to participate (Dillman, Smyth & Christian 2014), the absence of local ASF cases may result in lower disease awareness and reduced engagement with ASF-related communications (Jurado et al. 2018), psychological distance from the threat may diminish perceived personal relevance, a known predictor of survey participation in agricultural health studies (Brewer et al. 2007; Weinstein 1989) and farmers facing active threats demonstrate heightened information-seeking behaviour and greater engagement with research initiatives addressing their immediate challenges (Hernández-Jover et al. 2012; Mankad 2016; Racicot et al. 2011).

A census of pigs was conducted on farms included in the study to quantify the number of pig farmers in the selected regions of Gauteng. However, because of COVID-19 restrictions and voluntary participation of farmers, a full census of the areas was not completed. It is recommended that a comprehensive census be conducted to determine the approximate number of pigs and/or farms in each municipality, as some farmers reported recently establishing piggeries. The demographic profile of pig farmers in Gauteng aligns with trends observed across SA, where older individuals dominate smallholder pig production (Mokoele et al. 2014). While younger people often prefer formal employment, their engagement in pig farming could address unemployment and enhance productivity, given their adaptability to innovative management practices (Madzimure et al. 2012). Male farmers predominate in the sector, which is consistent with prior national studies (Matabane et al. 2015).

Emerging farmers represent a significant proportion of smallholder pig producers, highlighting the need for clear classification criteria to avoid potential bias in identifying farmer groups (Zantsi, Greyling & Vink 2019). Across all production systems, biosecurity practices are crucial to mitigating the risk of exposure to ASF. Permanent housing reduces exposure to disease, whereas unfenced or free-range systems increase vulnerability (Maoba, Manyakanyaka & Mamaregane 2022; Penrith 2013). The movement of pigs – particularly for trade or during outbreaks – remains a key factor in ASF transmission, necessitating regulatory measures such as movement permits and strict monitoring of auctions (Dione et al. 2016; Olugasa & Ijagbone 2007; Van Rensburg, Penrith & Etter 2022).

Knowledge gaps regarding ASF symptoms and biosecurity measures exacerbate disease risks. Limited awareness delays early detection, facilitating outbreaks (Aliro et al. 2022; Arvidsson et al. 2022; Casal et al. 2007). As described in Uganda (Dione et al. 2020), most of the training efforts in SA are often sporadic and project-specific, with no systematic evaluation of effectiveness. Evidence from Uganda suggests that increased awareness correlates with improved management practices, underscoring the importance of continuous farmer education (Nantima et al. 2015).

Effective hygiene and disinfection are pivotal in controlling ASF, particularly in the absence of a commercial vaccine. Practices such as routine cleaning of piggeries, handwashing, changing boots, and the use of footbaths have been shown to reduce biosecurity risks (Kouam, Jacouba & Moussala 2020; Maoba et al. 2022). Cleaning and disinfection of equipment and transport vehicles is especially important given the virus’s environmental resilience in organic material (Gao et al. 2023). Comparisons with regions lacking such practices, such as Tanzania’s Southern Highlands, demonstrate a direct link between poor hygiene and high ASF incidence (Fasina et al. 2020).

Feeding strategies also influence ASF risk. Use of untreated swill containing pork products is a well-documented transmission route (Nantima et al. 2015; Van Rensburg et al. 2020a). Economic constraints have led some farmers to supplement commercial feed with alternative sources, highlighting the need for affordable, safe feeding practices. Similarly, the use of pig waste as fertiliser can contribute to ASF spread if improperly managed, which is a common concern in integrated crop-livestock systems (Kabuuka et al. 2014).

Although in this study slaughtering has not been thoroughly investigated, the few farmers who reported slaughter did mention slaughtering at home and at abattoirs. Food safety may be jeopardised by improper slaughtering facilities and methods used in the unorganised sector (Rani et al. 2017). Meat from smallholder or backyard slaughter is often tainted and unsanitary. *The Meat Safety Act No. 40 of 2000*’s rules, which are intended to safeguard consumer health, are not always followed in the informal sector (Rani et al. 2017). Meat safety issues related to handling meat during distribution continue to be a source of worry, even if these standards are implemented in the official sector. Safe handling and disposal of sick or deceased pigs is essential to prevent virus dissemination. Isolation, veterinary consultation, and supervised disposal – including burial, incineration or burning – are recommended to minimise environmental contamination (Bellini, Rutili & Guberti 2016). Inappropriate handling poses a high risk of indirect transmission and compromises biosecurity.

The multiple comparison adjustment (Online Appendix 1, Table 1-OA1) demonstrates most of the biosecurity practice differences reported in the original data (Table 2-OA1, Table 3-OA1, and Table 4-OA1) may be false positives (Type I errors) arising from conducting multiple statistical tests without adjustment. The phenomenon of ‘multiple testing’ is well-established. When conducting 31 tests at α = 0.05, it is expected that ~1.5 of the tests will be significant by chance alone even if no true differences exist (Sterne & Smith 2001).

The key finding is that handwashing behaviour varies significantly according to ASF status, displaying a paradoxical pattern: farmers in ASF-free areas exhibit better hand hygiene than those in HRA without prior ASF experience. This counterintuitive finding warrants further investigation and suggests that factors beyond proximity to ASF outbreaks drive biosecurity adoption.

Overall, the study underscores that management practices, farmer knowledge and resource limitations critically shape ASF risk in smallholder systems. Even low-cost interventions, such as footbaths, routine cleaning and controlled pig movement, can substantially mitigate outbreak risk. These findings reinforce that preventive biosecurity remains the cornerstone of ASF control. The potential availability of vaccines would complement, rather than replace, sound biosecurity practices, as vaccination programmes are most effective in healthy, well-managed herds. This further underscores the importance of targeted education, accessible infrastructure and regulatory oversight in safeguarding pig health.

## Conclusion

This study indicated that fewer biosecurity practices were implemented in HRA areas, posing a potential risk of ASF spreading into Gauteng. Knowledge gaps are a plausible explanation for poor biosecurity adoption that requires verification through targeted follow-up research. This implies that farmers in Gauteng require training on ASF disease symptoms and piggery management, encouragement to practise safe feeding and adoption of appropriate husbandry practices. The research offers valuable insight into stakeholder engagement regarding disease risk perception, with higher engagement with populations at high risk for ASF outbreaks, which could lead to enhanced practical applicability of the biosecurity and risk management findings. This context-specific focus provides a possible model for outbreak-prone regions, although it limits generalisability. In addition, this study provides baseline data for Gauteng-specific policy development and describes a framework that other provinces can adapt.

## References

[CIT0001] Aliro, T., Chenais, E., Odongo, W., Okello, D.M., Masembe, C. & Stahl, K., 2022, ‘Prevention and control of African swine fever in the smallholder pig value chain in northern Uganda: Thematic analysis of stakeholders’ perceptions’, *Frontiers in Veterinary Science* 8, 707819. 10.3389/fvets.2021.707819 35097036 PMC8793068

[CIT0002] Arvidsson, A., Fischer, K., Hansen, K., Sternberg-Lewerin, S. & Chenais, E., 2022, ‘Diverging discourses: Animal health challenges and veterinary care in northern Uganda’, *Frontiers in Veterinary Science* 9, 773903. 10.3389/fvets.2022.773903 35359673 PMC8960384

[CIT0003] Asambe, A., Sackey, A.K.B. & Tekdek, L.B., 2019, ‘Sanitary measures in piggeries, awareness, and risk factors of African swine fever in Benue State, Nigeria’, *Tropical Animal Health and Production* 51(4), 997–1001. 10.1007/s11250-018-1764-7 30569230 PMC6469624

[CIT0004] Bellini, S., Rutili, D. & Guberti, V., 2016, ‘Preventive measures aimed at minimizing the risk of African swine fever virus spread in pig farming systems’, *Acta Veterinaria Scandinavica. BioMed Central* 58(1), 1–10. 10.1186/s13028-016-0264-x PMC5129245 27899125

[CIT0005] Brewer, N.T., Chapman, G.B., Gibbons, F.X., Gerrard, M., Mccaul, K.D. & Weinstein, N.D., 2007, ‘Meta-analysis of the relationship between risk perception and health behavior: The example of vaccination’, *Health Psychology* 26(2), 136–145. 10.1037/0278-6133.26.2.136 17385964

[CIT0006] Brown, C.C., Pan, D. & Wiersma, G., 2015. ‘Advanced data analysis: From Excel pivot tables to Microsoft Access’, in B.R. Bernhardt, L.H. Hinds & K.P. Strauch (eds.), JSTOR Where do we go from here? Proceedings of the Charleston Library Conference, Charleston, South Carolina, November 4–7, 2015, Purdue University Press, Grand Dragon West Lafayette, pp. 571–600.

[CIT0007] Brown, V.R. & Bevins, S.N., 2018, ‘A review of African swine fever and the potential for introduction into the United States and the possibility of subsequent establishment in feral swine and native ticks’, *Frontiers in Veterinary Science* 5, 11. 10.3389/fvets.2018.00011 29468165 PMC5808196

[CIT0008] Casal, J., De Manuel, A., Mateu, E. & Martin, M., 2007, ‘Biosecurity measures on swine farms in Spain: Perceptions by farmers and their relationship to current on-farm measures’, *Preventive Veterinary Medicine* 82(1–2), 138–150. 10.1016/j.prevetmed.2007.05.012 17590460

[CIT0009] Cochran, W.G., 1963. *Sampling techniques*, 2nd edn., John Wiley and Sons, Inc, New York, NY.

[CIT0010] Department of Agriculture, Forestry and Fisheries (DAFF), 2018, *Veterinary procedural notice: Biosecurity standards for pig compartments*, Department of Agriculture, Forestry and Fisheries, South Africa, Pretoria, viewed 27 July 2020, from https://www.scribd.com/document/826775653/DRAFT-African-Swine-Fever-VPN-for-consultation-2018-05

[CIT0011] Dillman, D.A., Smyth, J.D. & Christian, L.M., 2014, *Internet, phone, mail, and mixed mode surveys: The tailored design method*, 4th edn., Wiley, Hoboken, NJ.

[CIT0012] Dione, M., Ouma, E., Opio, F., Kawuma, B. & Pezo, D., 2016, ‘Qualitative analysis of the risks and practices associated with the spread of African swine fever within the smallholder pig value chains in Uganda’, *Preventive Veterinary Medicine* 135(1), 102–112. 10.1016/j.prevetmed.2016.11.001 27931922

[CIT0013] Dione, M.M., Dohoo, I., Ndiwa, N., Poole, J., Ouma, E., Amia, W.C. et al., 2020, ‘Impact of participatory training of smallholder pig farmers on knowledge, attitudes, and practices regarding biosecurity for the control of African swine fever in Uganda’, *Transboundary and Emerging Diseases* 67(6), 2482–2493. 10.1111/tbed.13587 32311216 PMC7754142

[CIT0014] Dixon, L.K., Stahl, K., Jori, F., Vial, L. & Pfeiffer, D.U., 2020, ‘African swine fever epidemiology and control’, *Annual Review of Animal Biosciences* 8, 221–246. 10.1146/annurev-animal-021419-083741 31743062

[CIT0015] Fasina, F.O., Kissinga, H., Mlowe, F., Mshang’a, S., Matogo, B., Mrema, A. et al., 2020, ‘Drivers, risk factors and dynamics of African swine fever outbreaks, Southern Highlands, Tanzania’, *Pathogens* 9(3), 155. 10.3390/pathogens9030155 32106538 PMC7157628

[CIT0016] Fasina, F.O., Mokoele, J.M., Spencer, B.T., Van Leengoed, L.A., Bevis, Y. & Booysen, I., 2015, ‘Spatio-temporal patterns and movement analysis of pigs from smallholder farms and implications for African swine fever spread, Limpopo province, South Africa’, *Onderstepoort Journal of Veterinary Research* 82(1), 1–11. 10.4102/ojvr.v82i1.795 PMC6238709 26842362

[CIT0017] Fujita, T., 2004, ‘Zoning and regulatory for animal disease control’, in 11th International Conference of the Association of Institutions for Tropical Veterinary Medicine and 16th Veterinary Association Malaysia Congress, August 23–27, Sunway Pyramid Convention Centre, Petaling Jaya, pp. 14–16, OIE Regional Representation for Asia and the Pacific.

[CIT0018] Gao, Y., Nielsen, L.H., Boklund, A.E., De Jong, M.C.M. & Alban, L., 2023, ‘SWOT analysis of risk factors associated with introduction of African swine fever through vehicles returning after export of pigs’, *Frontiers in Veterinary Science* 9, 1049940. 10.3389/fvets.2022.1049940 36686159 PMC9846816

[CIT0019] Groves, R.M., Singer, E. & Corning, A., 2000, ‘Leverage-saliency theory of survey participation: Description and an illustration’, *Public Opinion Quarterly* 64(3), 299–308. 10.1086/317990 11114270

[CIT0020] Hernández-Jover, M., Taylor, M., Holyoake, P. & Dhand, N., 2012, ‘Pig producers’ perceptions of the Influenza Pandemic H1N1/09 outbreak and its effect on their biosecurity practices in Australia’, *Preventive Veterinary Medicine* 106(3–4), 284–294. 10.1016/j.prevetmed.2012.03.008 22487168

[CIT0021] Jurado, C., Martínez-Avilés, M., De La Torre, A., Štukelj, M., De Carvalho, F.H.C., Cerioli, M. et al., 2018, ‘Relevant measures to prevent the spread of African swine fever in the European Union Domestic Pig Sector’, *Frontiers in Veterinary Science* 5, 77. 10.3389/fvets.2018.00077 29713637 PMC5912175

[CIT0022] Kabuuka, T., Kasaija, P.D., Mulindwa, H., Shittu, A., Bastos, A.D. & Fasina, F.O., 2014, ‘Drivers and risk factors for circulating African swine fever virus in Uganda, 2012–2013’, *Research in Veterinary Science* 97(2), 218–225. 10.1016/j.rvsc.2014.07.001 25066802

[CIT0023] Kouam, M.K., Jacouba, M. & Moussala, J.O., 2020, ‘Management and biosecurity practices on pig farms in the Western Highlands of Cameroon (Central Africa)’, *Veterinary Medicine and Science* 6(1), 82–91. 10.1002/vms3.211 31682081 PMC7036310

[CIT0024] Madzimure, J., Chimonyo, M., Zander, K.K. & Dzama, K., 2012, ‘Potential for using indigenous pigs in subsistence-oriented and market-oriented small-scale farming systems of Southern Africa’, *Tropical Animal Health and Production* 45(1), 135–142. 10.1007/s11250-012-0184-3 22639035

[CIT0025] Mankad, A., 2016, ‘Psychological influences on biosecurity control and farmer decision-making. A review’, *Agronomy for Sustainable Development* 36, 40. 10.1007/s13593-016-0375-9

[CIT0026] Maoba, S., Manyakanyaka, B. & Mamaregane, P.A., 2022, ‘Evaluation of biosecurity practices in smallholder pig production systems in Midvaal local municipality, Gauteng province’, *Applied Animal Husbandry & Rural Development* 15, 1–9.

[CIT0027] Matabane, M.B., Nethenzheni, P., Thomas, R., Netshirovha, T.R., Norris, D., Nephawe, K.A. et al., 2015, ‘Status of the smallholder pig farming sector in Gauteng Province of South Africa’, *Applied Animal Husbandry & Rural Development* 8(1), 19–25.

[CIT0028] Mokoele, J.M., Spencer, B.T., Van Leengoed, L.A. & Fasina, F.O., 2014, ‘Efficiency indices and indicators of poor performance among emerging small-scale pig farmers in the Limpopo Province, South Africa’, *Onderstepoort Journal of Veterinary Research* 81(1), 1–12. 10.4102/ojvr.v81i1.774 25685963

[CIT0029] Montgomery, R.E., 1921, ‘On a form of swine fever occurring in British East Africa (Kenya Colony)’, *Journal of Comparative Pathology and Therapeutics* 34(1), 159–191. 10.1016/S0368-1742(21)80031-4

[CIT0030] Munzhelele, P., Oguttu, J.W. & Fasina, F.O., 2016, ‘Is a 10-sow unit economically sustainable? A profitability assessment of productivity amongst small-holder pig farmers, Mpumalanga, South Africa’, *Onderstepoort Journal of Veterinary Research* 83(1), 1011. 10.4102/ojvr.v83i1.1011 27247064 PMC6238787

[CIT0031] Nantima, N., Ocaido, M., Ouma, E., Davies, J., Dione, M., Okoth, E. et al., 2015, ‘Risk factors associated with occurrence of African swine fever outbreaks in smallholder pig farms in four districts along the Uganda-kenya border’, *Tropical Animal Health and Production* 47(3), 589–595. 10.1007/s11250-015-0768-9 25616986

[CIT0032] Nhamo, L., Rwizi, L., Mpandeli, S., Botai, J., Magidi, J., Tazvinga, H. et al., 2021, ‘Urban nexus and transformative pathways towards a resilient Gauteng City-Region, South Africa’, *Cities* 116, 103266. 10.1016/j.cities.2021.103266 37674556 PMC7615023

[CIT0033] Olugasa, B.O. & Ijagbone, I.F., 2007, ‘Pattern of spread of African swine fever in south-western Nigeria, 1997–2005’, *Veterinaria Italiana* 43(3), 621–628. 20422541

[CIT0034] Penrith, M.L., 2013, ‘History of “swine fever” in southern Africa’, *Journal of the South African Veterinary Association* 84(1), 1–6. 10.4102/jsava.v84i1.1106

[CIT0035] Penrith, M.L., 2020, ‘Current status of African swine fever’, *CABI Agriculture and Bioscience* 1(1), 1–26. 10.1186/s43170-020-00011-w

[CIT0036] Racicot, M., Venne, D., Durivage, A. & Vaillancourt, J.P., 2011, ‘Description of 44 biosecurity errors while entering and exiting poultry barns based on video surveillance in Quebec, Canada’, *Preventive Veterinary Medicine* 100, 193–199. 10.1016/j.prevetmed.2011.04.011 21605922

[CIT0037] Rani, Z., Hugo, A., Hugo, C., Vimiso, P. & Muchenje, V., 2017, ‘Effect of post-slaughter handling during distribution on microbiological quality and safety of meat in the formal and informal sectors of South Africa: A review’, *South African Journal of Animal Science* 47, 255–267. 10.4314/sajas.v47i3.2

[CIT0038] Sánchez-Cordón, P.J., Montoya, M., Reis, A.L. & Dixon, L.K., 2018, ‘African swine fever: A re-emerging viral disease threatening the global pig industry’, *Veterinary Journal* 233, 41–48. 10.1016/j.tvjl.2017.12.025 29486878 PMC5844645

[CIT0039] Stats SA, 2016, *Community survey 2016 agricultural households*, Community Survey, Statistics South Africa, Pretoria, viewed 31 August 2020, from http://www.statssa.gov.za/publications/03-01-05/03-01-052016.pdf.

[CIT0040] Sterne, J.A.C. & Smith, G., 2001, ‘Sifting the evidence – What’s wrong with significance tests?’, *British Medical Journal* 322(7280), 226–231. 10.1136/bmj.322.7280.226 11159626 PMC1119478

[CIT0041] Thrusfield, M., 2005, *Veterinary epidemiology*, 2nd edn., p. 183, Blackwell Science, Oxford.

[CIT0042] Van Rensburg, L., Etter, E., Heath, L., Penrith, M.L. & Van Heerden, J., 2020a, ‘Understanding African swine fever outbreaks in domestic pigs in a sylvatic endemic area: The case of the South African controlled area between 1977–2017’, *Transboundary and Emerging Diseases* 67(6), 2753–2769. 10.1111/tbed.13632 32438525

[CIT0043] Van Rensburg, L., Penrith, M.L. & Etter, E., 2022, ‘Prioritisation of provinces for African swine fever intervention in South Africa through decision matrix analysis’, *Pathogens* 11(2), 135. 10.3390/pathogens11020135 35215079 PMC8880338

[CIT0044] Van Rensburg, L., Van Heerden, J., Heath, L.E., Rametse, T., Etter, E.M. & Penrith, M.L., 2020b, ‘Investigation of African swine fever outbreaks in pigs outside the controlled areas of South Africa, 2012–2017’, *Journal of the South African Veterinary Association* 91(1), 1–9. 10.4102/jsava.v91i0.1997 PMC7433221 32787419

[CIT0045] Weinstein, N.D., 1989, ‘Effects of personal experience on self-protective behavior’, *Psychological Bulletin* 105(1), 31–50. 10.1037/0033-2909.105.1.31 2648439

[CIT0046] Zantsi, S., Greyling, J.C. & Vink, N., 2019, ‘Towards a common understanding of “emerging farmer” in a South African context using data from a survey of three district municipalities in the Eastern Cape Province’, *South African Journal of Agricultural Extension* 47(2), 81–93. 10.17159/2413-3221/2019/v47n2a505

